# Altered Pancreatic Growth and Insulin Secretion in WSB/EiJ Mice

**DOI:** 10.1371/journal.pone.0088352

**Published:** 2014-02-05

**Authors:** Maggie M. Ho, Xiaoke Hu, Subashini Karunakaran, James D. Johnson, Susanne M. Clee

**Affiliations:** 1 Department of Cellular and Physiological Sciences, Life Sciences Institute, University of British Columbia, Vancouver, British Columbia, Canada; 2 Department of Surgery, Life Sciences Institute, University of British Columbia, Vancouver, British Columbia, Canada; CRCHUM-Montreal Diabetes Research Center, Canada

## Abstract

These data suggest that insulin secretion in WSB mice is blunted specifically *in vivo*, either due to a reduced insulin requirement and/or due to factors that are absent or destroyed *in vitro*. These studies also highlight the role of post-natal growth in determining adult β-cell mass. Mice are important animal models for the study of metabolic physiology and the genetics of complex traits. Wild-derived inbred mouse strains, such as WSB/EiJ (WSB), are unrelated to the commonly studied mouse strains and are valuable tools to identify novel genes that modify disease risk. We have previously shown that in contrast to C57BL/6J (B6) mice, WSB mice fed a high fat diet do not develop hyperinsulinemia or insulin resistance, and had nearly undetectable insulin secretion in response to an intraperitoneal glucose challenge. As hyperinsulinemia may drive obesity and insulin resistance, we examined whether defects in β-cell mass or function could contribute to the low insulin levels in WSB mice. In young WSB mice, β-cell mass was similar to B6 mice. However, we found that adult WSB mice had reduced β-cell mass due to reduced pancreatic weights. Pancreatic sizes were similar between the strains when normalized to body weight, suggesting their pancreatic size is appropriate to their body size in adults, but overall post-natal pancreatic growth was reduced in WSB mice compared to B6 mice. Islet architecture was normal in WSB mice. WSB mice had markedly increased insulin secretion from isolated islets *in vitro*. These data suggest that insulin secretion in WSB mice is blunted specifically *in vivo*, either due to a reduced insulin requirement and/or due to factors that are absent or destroyed *in vitro*. These studies suggest that WSB mice may provide novel insight into mechanisms regulating insulin secretion and also highlight the role of post-natal growth in determining adult β-cell mass.

## Introduction

Type 2 diabetes (T2D) develops when the β-cells of the pancreas cannot produce enough insulin to meet the body’s demands, which are increased when an individual becomes resistant to insulin. Thus β-cell dysfunction is a key component of T2D [1]. Both factors affecting β-cell mass and insulin secretion are important [1]. However, the molecular determinants of these processes are incompletely understood. T2D has a strong genetic component [2] that could provide clues about the critical factors affecting T2D risk. Indeed many of the factors that have recently been identified in the genome-wide association studies (GWAS) have been suggested to affect properties of β-cells [3], [4]. While the arrays used in the GWAS may contain thousands of SNPs accounting for up to 50% of the variation in T2D risk [5], only ∼60 of these SNPs have been identified to date, and the majority of the genetic factors contributing to T2D are still unknown [4].

Studies of inbred mouse strains have provided insight into T2D pathogenesis [6], and genetic analysis of these strains has identified genetic factors that could affect T2D risk [7]–[11]. Genetic differences between inbred strains are like genetic differences between human individuals, but unlimited numbers of these individuals can be produced for study. However, the classical inbred mouse strains, such as C57BL/6J (B6), that have largely been used for study so far share large portions of their genome due to their common ancestry [6], [12], [13]. This restricts the phenotypes observed, and hinders genetic studies to discover underlying molecular contributors. To overcome this limitation, new strains have recently been generated from wild-caught mice. These wild-derived inbred strains are thus unrelated to the commonly studied strains, and over 75% of the known genetic variation in mice is found only in these newer wild derived inbred strains, such as WSB/EiJ (WSB) [12]. Because of this increased genetic diversity, WSB mice were included in several large-scale genetic and genomic projects such as the Collaborative Cross and the Sanger Mouse Genomes project [12], [14]–[16]. As such, it is important to define and refine the phenotypes associated with the genome of this and other wild-derived inbred strains, to maximize the power and informativeness of genetic screens by enabling researchers to test the most specific phenotypes within genetic mapping studies including them.

Previously, we examined whether the novel genetic variation present in WSB mice affects diabetes and obesity-related traits [17]. In contrast to B6 mice, we found that fasting insulin levels did not increase in high fat-fed WSB mice. WSB mice fed a high fat diet remained lean throughout, and although similar at 6 weeks of age, WSB mice were more insulin sensitive than B6 mice from 10 weeks of age onwards. Increased fasting insulin can promote both obesity and insulin resistance [18]. In response to an intraperitoneal glucose challenge, WSB mice had minimal first phase (prior to 15 min) and undetectable second phase insulin secretion *in vivo* [17]. The goal of the present studies was to examine physiological mechanisms that could contribute to the low insulin phenotype of WSB mice, to identify inherent phenotypes of this strain amenable to future genetic analysis. Here we found that WSB mice have reduced post-natal pancreatic growth that results in reduced β-cell mass compared to adult B6 mice. In contrast to the low glucose-stimulated insulin secretion *in vivo*, insulin secretion was markedly elevated from islets *in vitro*, suggesting a release from factors that control insulin secretion *in vivo*.

## Methods

### Animals

Male C57BL/6J and WSB/EiJ mice were housed in an environmentally controlled facility with twelve hour light/dark cycles (7 am–7 pm). The mice were fed either a standard rodent chow (LabDiet 5010, Jamieson’s Pet Food Distributors, Delta, BC, Canada) or a diet containing sixty percent calories from fat (lard) and twenty percent calories from sugar (sucrose and maltodextrin, D12492, Research Diets, New Brunswick, NJ). The diets were provided from weaning, and the mice had unlimited food and water access throughout the studies. The mice were euthanized at the specified ages by CO_2_ asphyxiation followed by cervical dislocation and the rapid removal of the pancreas for analysis. All procedures were performed according to Canadian Council on Animal Care guidelines and approved by the UBC Committee on Animal Care.

### Pancreatic Islet Isolation and Perifusion

Islets were isolated from C57BL/6J and WSB/EiJ mice (6–7 weeks of age) by collagenase digestion and purified using the modified filtration method of Salvalaggio, as described [19], [20]. The islets were subsequently handpicked and incubated in 11 mM glucose-containing RPMI media (Invitrogen, Burlington, ON, Canada) overnight at 37°C with 5% CO_2_. The next day, one hundred size-matched islets from individual mice of the two strains were picked into individual chambers for perifusion, as previously described [21]. Perfusate solutions were comprised of Krebs Ringer Bicarbonate buffer containing 3 mM glucose (basal), 20 mM glucose, or basal glucose plus 30 mM KCl, and were flowed through the chambers at a rate of 1 mL per minute with 20–30 minutes for each secretagogue, separated by a period at basal glucose. Perfusate fractions were collected every 5 min and insulin measured by ELISA, as described [22].

### Pancreatic Insulin Content

Following euthanasia at 6–7 weeks of age, pancreata from C57BL/6J and WSB/EiJ mice were dissected, weighed and rapidly placed in ice-cold acid ethanol. Pancreata were minced and stored at −20°C for insulin extraction, as previously described [8]. Insulin was measured by ELISA [22].

### Immunohistochemistry

C57BL/6J and WSB/EiJ pancreata were collected at the specified age, weighed, fixed overnight in paraformaldehyde, infiltrated overnight in 30% sucrose, and frozen in Neg50 media (Fisher Scientific, Toronto ON, Canada). Ten micron cryosections (each a minimum of 300 microns apart containing tissue from the head and tail of the pancreas) were obtained at multiple levels through the pancreas for immunofluorescent staining of insulin and glucagon, as previously described [8]. Stained tissue sections (n = 2–3 per mouse) were imaged on a fluorescent microscope with automated scanning of the entire section (Zeiss Axiovert 100, Carl Zeiss Canada, Toronto ON, Canada). Images of the tissue section were reassembled, and insulin, glucagon and total tissue (DAPI) staining areas were measured using Slidebook (Intelligent Imaging Innovations, Denver CO, USA). Samples were processed in several batches, with mice from each strain and diet in each batch.

For vascular staining, adjacent sections were stained for insulin, as above, and endothelial cells (rat anti-mouse CD31, BD Pharmingen #550274, 1∶50). Islets were identified in the re-assembled sections using the boundaries defined by the insulin staining, and the total CD31-positive and insulin positive areas within these regions of interest determined.

### Statistical Analysis

Statistical analysis was performed by ANOVA with post-hoc Tukey pair-wise comparisons or two-way ANOVA (islet size distributions) followed by Bonferroni pair-wise comparisons between the genotypes in each size group, as appropriate. Data are shown as mean±standard error. P-values <0.05 were considered significant.

## Results

### Islet Architecture and β-cell Mass

Our previous studies found that unlike B6 mice, fasting plasma insulin levels that did not increase with age or high fat feeding in WSB mice, and that they exhibited minimal glucose-stimulated insulin secretion in response to an intraperitoneal glucose challenge *in vivo* [17]. To determine whether WSB mice have a defect in islet architecture or reduced β-cell mass that could contribute to these low insulin levels after chronic high fat feeding, we performed immunohistochemical morphometric analyses of islets from adult chow and high fat-fed WSB and B6 mice at 20 weeks of age. At this age fasting insulin levels were markedly increased in high fat-fed B6 mice, while levels in chow and high fat-fed WSB mice were not different from those in chow-fed B6 mice [17].

**Figure 1:**
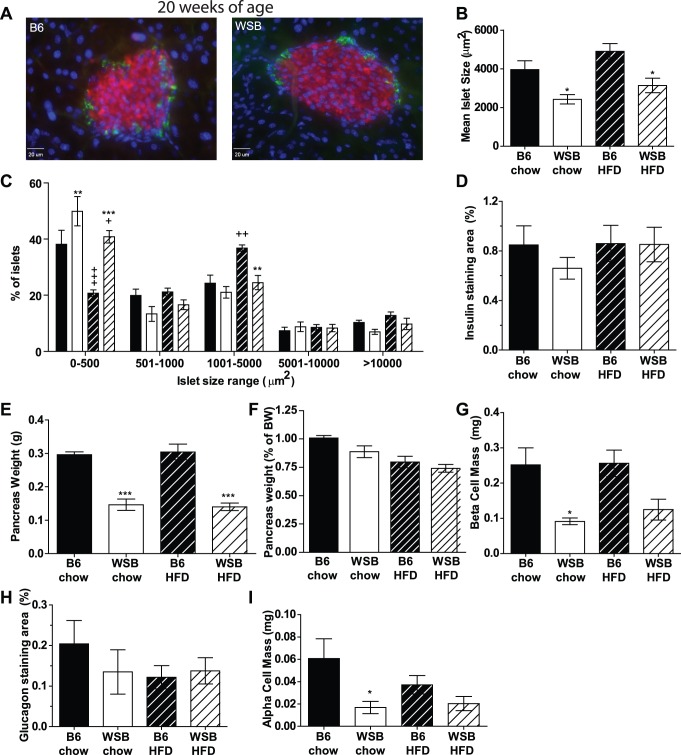
Islet structure and size, β-cell mass, and α-cell mass at twenty weeks of age. A. Representative islets from B6 (left) and WSB (right) mice fed the chow diet. Insulin is stained red, glucagon green, and cell nuclei are marked by DAPI staining (blue). No alteration to islet structures was found in islets from the high fat-fed mice. B. The average cross-sectional areas of all islets found in each mouse were determined then averaged for each strain (average number of islets per mouse examined: B6 chow- 167, B6 HFD 187, WSB chow 129, WSB HFD 171). C. Distribution of islet sizes in B6 (black; chow-fed solid, high fat diet (HFD)-fed striped) and WSB mice (white; chow-fed solid, high fat diet (HFD)-fed striped). In the upper group, although there was substantial overlap in the sizes, B6 mice tended to have larger maximal islet sizes (both diets). D. Percent of pancreatic tissue area stained for insulin. E. Pancreatic weight of mice used in these experiments. F. Pancreatic weights normalized to total body weight in chow and high fat-fed WSB and B6 mice. G. β-cell mass, calculated as the % staining area multiplied by the pancreatic weight. H. Percent of pancreatic area stained for glucagon. I. α-cell mass calculated as for β-cell mass. (n = 5 mice per strain per diet). *p<0.05, **p<0.01, ***p<0.001 vs. B6 mice consuming the same diet. +p<0.05, +++p<0.001 vs. chow-fed of the same strain.

Islet architecture appeared normal in WSB mice, with a solid core of insulin-producing cells surrounded by glucagon-producing cells (Figure 1A). Mean islet sizes in WSB mice were significantly reduced compared to B6 mice (Figure 1B). When examining the distribution of islet sizes, WSB mice had an increased proportion of smaller islets compared to B6 mice on either diet (Figure 1C). Also, in contrast to B6 mice fed the high fat diet, which had an increase in larger sized islets compared to chow-fed B6 mice, no significant effects of high fat feeding on islet sizes were observed in WSB mice (Figure 1C). However, despite these modest differences in islet sizes, the total insulin staining area as a percentage of pancreatic area was not significantly different between the two strains (Figure 1D), suggesting the reduced islet sizes may be compensated for by an increase in the number of islets.

Notably, the pancreas weight of WSB mice was significantly lower than that of B6 mice (Figure 1E). WSB mice are smaller than B6 mice [17], and as such it might be expected that their pancreas is smaller. When normalized for total body weight, there were no differences in pancreatic size between the strains (Figure 1F), suggesting that the reduction in pancreatic size is appropriate for their smaller body size. However, given this lower pancreatic size, total β-cell mass was significantly reduced in chow-fed WSB mice compared to B6 mice (Figure 1G). Similar trends were observed in the high fat-fed mice, although these did not reach statistical significance. Likewise, there was no significant difference in glucagon-staining areas per pancreatic tissue area between the strains (Figure 1H), but total α-cell mass was lower in chow-fed WSB mice compared to B6 mice (Figure 1I).

**Figure 2:**
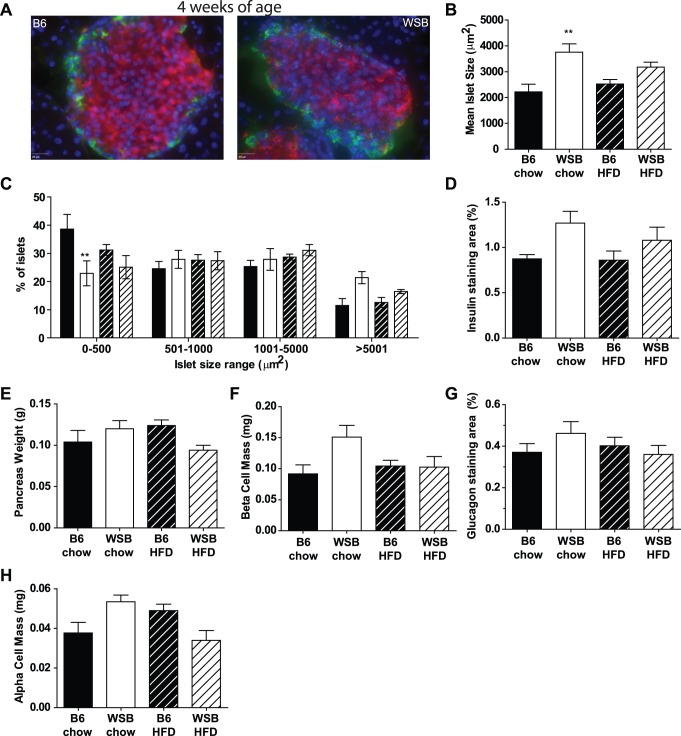
Islet structure and size, β-cell mass, and α-cell mass in young WSB mice. Studies were performed on mice at 4 weeks of age. A. Representative islets from B6 (left) and WSB (right) mice. Insulin (red), glucagon (green) and DNA (blue) are stained. B. Mean islet sizes (average number of islets per mouse examined: B6 chow- 166, B6 HFD 130, WSB chow 108, WSB HFD 115). C. Islet size distribution of B6 (black; chow solid, high fat diet (HFD) striped) and WSB (white; chow solid, high fat diet white striped) mice. The range of islet sizes observed was similar in all groups. D. Percent of pancreatic area stained for insulin. E. Pancreas weight for stained tissues. F. β-cell mass. G. Percent of pancreatic area stained for glucagon. H. α-cell mass. (n = 5 mice per strain per diet). **p<0.01 vs. B6 mice consuming the same diet.

At 20 weeks of age, WSB mice are more insulin sensitive than B6 mice [17]. To determine whether these differences in β-cell mass are inherent to WSB mice, or whether they resulted from differences in response to aging and the high fat diet such as increasing insulin resistance in the B6 mice, we focused the remainder of our analyses on young mice, prior to the time when we detected differences in insulin sensitivity [17]. Thus, we re-examined β-cell mass at four weeks of age. Again, islet architecture appeared normal in WSB mice (Figure 2A). In contrast to what was observed at 20 weeks of age, however, islets of WSB mice were larger than those of B6 mice (Figure 2B), suggesting WSB mice do not have a defect in the development of islets. At this age, chow-fed WSB mice had a lower percentage of small islets and tended to have a higher percentage of larger islets than chow-fed B6 mice (Figure 2C). Consistent with the increased islet sizes in chow-fed WSB mice at this age, the percentage of pancreatic area stained for insulin tended to be higher in WSB mice (Figure 2D). Although similar patterns were observed, no significant differences in islet areas were found in the high fat-fed mice. Pancreatic weights were not significantly different between the groups (Figure 2E) and in these young mice, there were no significant differences in β-cell or α-cell mass between the strains (Figure 2F–H).

**Figure 3:**
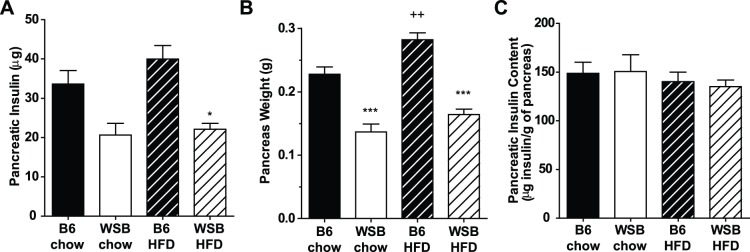
Whole pancreas insulin content. These studies were performed in young mice between 6 and 7 weeks of age. A. Total pancreatic insulin content. B. Pancreas weight. C. Pancreatic insulin content per gram of pancreas. (n = 5–6 WSB per diet, n = 10 B6 per diet) *p<0.05, ***p<0.001 vs. B6 mice consuming the same diet. ++p<0.01 vs. chow-fed mice of the same strain.

As β-cell mass is determined by staining area and does not account for the insulin content of the stained area, we also examined total pancreatic insulin content in young mice (6–7 weeks of age). Total pancreatic insulin contents were significantly lower in WSB mice (Figure 3A). However, when pancreatic insulin content was normalized to pancreatic weight (Figure 3B), there were no significant differences between the groups (Figure 3C), suggesting the difference resulted primarily due to differences in pancreatic size. Indeed, in examining the above data, we noticed that the strains differed in pancreatic weight depending on age. Pancreatic weights at 4 weeks of age were not different between the strains (Figure 2E). By 6–7 weeks of age, however, pancreatic weights more than doubled in B6 mice (Figure 3B) compared to 4 weeks of age (P<0.001), whereas in WSB mice they remained similar (chow) or increased slightly (high fat-fed, ∼50%, P<0.01), resulting in significantly higher weights in B6 mice compared to WSB mice. This difference in pancreatic weights between the strains at 6–7 weeks of age accounted for most of the difference that was evident at 20 weeks of age (Figure 1E), with pancreatic weight increasing between ∼7 and 20 weeks only in chow-fed B6 mice (P<0.001). At all three ages, insulin staining area or insulin content per amount of pancreatic tissue was similar between the strains, consistent with increased overall pancreatic growth in B6 mice.

### Insulin Secretion

As insulin secretion in response to an intraperitoneal glucose challenge was nearly undetectable in WSB mice [17], we examined the secretory function of islets from young (∼6 weeks of age) WSB mice by perifusion to determine whether WSB mice have impaired insulin secretion. This age was chosen over 4 weeks of age because WSB mice are small, and islet isolation was facilitated when the mice were a bit older. There were no differences in insulin sensitivity [17] or normalized insulin content between the strains at this age. To further remove any potential effects of high fat feeding, we focussed these studies on islets from chow-fed mice.

**Figure 4:**
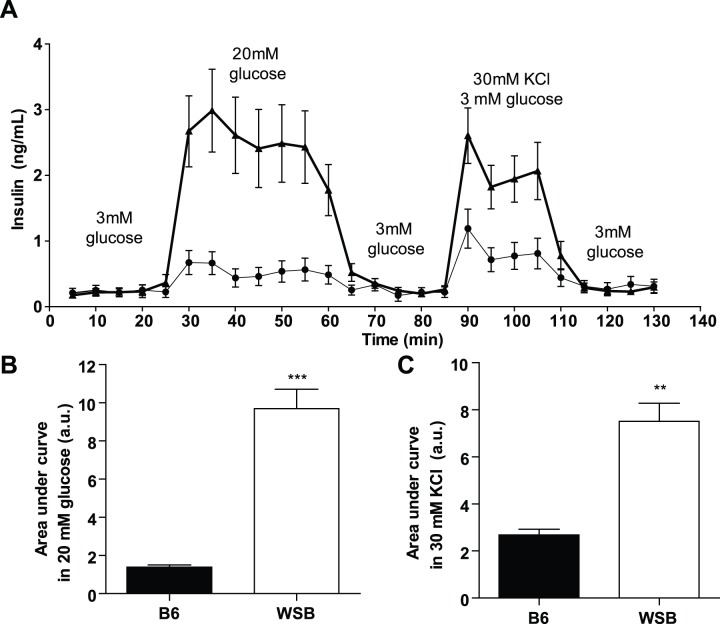
Insulin secretion in WSB mice. A, Insulin secretion response of size-matched B6 and WSB islets in 3 mM basal glucose, 20 mM glucose, and 30 mM KCl for the times indicated. Thin line represents B6 islets, Thick line represents WSB islets. B, Area between insulin secretion curve and average basal glucose insulin response when incubated with 20 mM glucose. C, Area between insulin secretion curve and average basal glucose insulin response when incubated with 30 mM potassium chloride in basal glucose. Perifusions were performed in ∼6 week old mice (n = 10 per strain). **p<0.01, ***p<0.001 vs. B6.

Surprisingly, insulin levels in the perfusates from WSB islets were ∼3 times higher than size-matched islets from B6 mice when stimulated with high glucose (Figure 4A). This resulted in a total of ∼7-fold more insulin released from WSB islets compared to B6 islets (Figure 4B). Typical first and second phase insulin secretion was observed in both strains. The amount of insulin secreted at basal glucose concentrations was similar between WSB and B6 mice, demonstrating that the WSB islets were not releasing their content in the absence of stimulation or due to the selection of larger islets from WSB mice. When insulin secretion was triggered with potassium chloride, insulin levels in the perfusate from WSB islets were similar to those achieved with high glucose, and roughly doubled relative to B6 islets (Figure 4A). It total, islets from WSB mice secreted nearly 3 times more insulin compared to B6 islets (Figure 4C). This suggested that although secretion in response to glucose stimulation *in vivo* is reduced [17], WSB β-cells were capable of secreting a high level of insulin.

**Figure 5:**
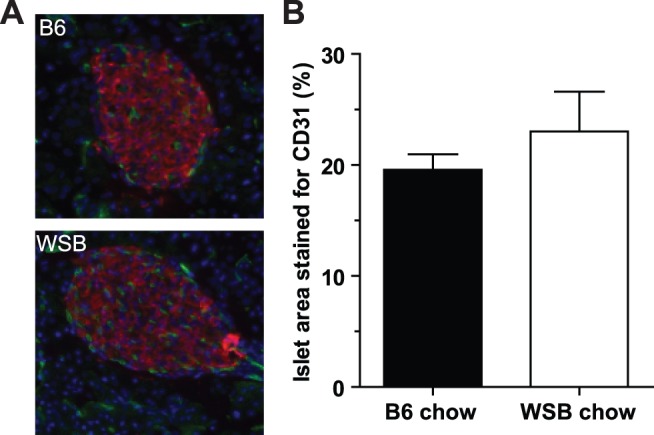
Islet vascularization at 4 weeks of age. A. Islet vascularization of chow-fed C57BL/6J (top) and WSB/EiJ (bottom) mice. Vasculature (CD31) is stained in green, while insulin is stained red to demarcate islet areas. B. Percent of islet area stained for CD31. (n = 5 per strain).

One critical factor that differs between these two situations is islet vascularization. Vascular density within islets has been shown to affect secretion *in vivo* [23]. To determine whether vascularization is reduced in WSB mice, we examined CD31 staining as a marker of endothelial cells in the islet [23]. The amount of islet vascularization (CD31 staining area within islets) was not different between WSB and B6 islets (Figure 5). This suggested that islet vascular density was likely not the factor affecting insulin secretion from WSB islets *in vivo*.

## Discussion

In previous studies, we found that compared to B6 mice, WSB mice maintained low fasting plasma insulin levels over time or with high fat feeding, remained insulin sensitive and secreted minor amounts of insulin in response to a glucose challenge *in vivo* [17]. While the low fasting insulin levels may be due to a reduced requirement for insulin due to their increased insulin sensitivity, it is also possible that reduced insulin production or secretion is what allows them to maintain insulin sensitivity [18]. Genetic studies are facilitated by the analysis of specific traits that arise due to genetic differences, as opposed to those that occur secondary to other changes. Thus, we examined whether WSB mice have alterations in β-cell mass or function that may contribute to these phenotypes. These studies revealed that WSB mice have reduced post-natal pancreatic growth that results in decreased β-cell mass in adults, and markedly enhanced insulin secretion from isolated islets *in vitro*.

Decreased β-cell proliferation is recognized as a mechanism by which β-cell mass may become insufficient to meet the body’s needs, and many studies have examined β-cell development and proliferation of adult β-cells, e.g. in response to insulin resistance [1], [24]–[26]. Shortly after weaning (4 weeks of age), WSB mice had similar or even increased islet areas and β-cell mass compared to B6 mice, suggesting that embryonic and early postnatal pancreatic development is not impaired in WSB mice. Despite the fact that WSB mice are ∼15–20% lighter than B6 mice at this age [17], they had similar pancreas weights. However, by 6–7 weeks of age, pancreatic weights were significantly lower in WSB mice compared to B6 mice. Whereas pancreatic weights increased 2.5 to 3-fold in B6 mice by 20 weeks of age, in WSB mice they increased only 20–50% during this timeframe. Yet islets from WSB mice did increase in size, with islet areas >10,000 µm^2^ by 20 weeks of age that were not apparent at 4 weeks of age, suggesting that postnatal islet growth does occur in WSB mice. At the older ages, insulin content and insulin staining areas per amount of pancreas were similar between the strains, suggesting a uniform change in both the endocrine and exocrine compartments. Adult WSB mice are smaller than B6 mice in terms of body weight and body length [17], and when pancreatic weights were normalized to body weight, they were similar between the strains (Figure 1F). Combined, these data suggest that the difference in pancreatic weights between the strains may be reflective of different post-natal growth trajectories between the strains.

β-cell mass has been shown to increase through development until roughly weaning, after which further expansion is thought to occur by proliferation, when required [24]. However, pancreatic weights have been shown to continue to increase until ∼7 weeks of age in mice [27]. Significant increases in pancreas weight post-weaning have been observed in several species [28], yet less is known about this process. Alterations, for example, in Wnt signaling can lead to markedly increased pancreatic weights when normalized to body weight [27], whereas defective prolactin signaling may also be a mechanism of reduced pancreatic growth affecting both acinar and exocrine tissue [29]. While factors affecting pancreatic development have been extensively studied, and while different mechanisms are known to affect β-cell proliferation during development and in adults [24], differences in post-natal pancreatic growth may be an under-appreciated determinant of adult β-cell mass.

We previously observed an apparent lack of secretory response to a glucose challenge *in vivo* in WSB mice [17]. We thus examined insulin secretion directly, and were surprised to find such a robust response of their islets to a glucose challenge *in vitro*. The perifusion studies were performed on young mice with hand-picked islets matched for size between WSB and B6 mice. The size range of the islets was similar between the strains, just with differences in their relative proportions. Furthermore, insulin secretion in basal glucose was similar between the strains. Thus, this degree of increased secretion is not likely simply due to the inclusion of larger islets in WSB mice relative to B6 mice. In young WSB mice although islets were on average larger, total insulin staining areas were similarly increased, consistent with no gross changes in islet number. However, the insulin content per pancreatic amount was similar, which suggests, if anything, reduced insulin content per islet in WSB mice at this age. These studies were performed on young, chow-fed mice, and are thus not complicated by high fat diet-induced changes in insulin secretion [31]. Thus, insulin secretion is clearly increased from WSB islets compared to B6 islets *in vitro*.

The increased insulin secretion from WSB islets was observed both in response to glucose and to depolarization with potassium, suggesting that it results from augmentation of pathways downstream of depolarization, e.g. granule trafficking [30], and/or islet or granule insulin content. The increased insulin release occurred immediately upon stimulation, with observable first and second phases, favouring release of an increased number of granules or an increased insulin content of those granules as potential explanations for the increased secretion. Our studies cannot distinguish between the possible mechanisms. Further detailed studies of granule biology are necessary to determine the mechanism(s) leading to increased insulin release from WSB islets.

The finding of increased insulin secretion from WSB islets *in vitro* appears to be discrepant with the blunted insulin secretory response *in vivo* [17]. However, in our previous work, insulin secretion was measured in response to an intraperitoneal glucose challenge, not by a more sensitive hyperglycemic clamp, and it is possible that we missed a short-lived peak of insulin secretion between sampling points. Insulin secretion *in vivo* was assessed in WSB mice at ages at which they were more insulin sensitive than the B6 controls, which suggests their insulin requirement was lower, and thus it would be expected that insulin secretion would be reduced. In contrast, these studies were performed on islets from chow-fed mice at 6 weeks of age, prior to measured differences in insulin sensitivity between the strains. However, although we did not detect differences in insulin sensitivity between the strains (even in chow-fed mice) at 6 weeks of age, because these were measured by intraperitoneal insulin tolerance test, it is possible we failed to detect more subtle, e.g. tissue-specific, diferences in insulin sensitivity at this age. Despite these caveats, the amount of insulin measured *in vivo* following a glucose challenge were negligible, suggesting that either WSB mice have miniscule requirements for insulin or that insulin secretion *in vivo* is blunted compared to the response observed *in vitro*.

There are several potential factors that may cause a difference in insulin secretion *in vivo* versus *in vitro*. Secretion *in vivo* could indeed be reduced due to the lower β-cell mass in adult WSB mice contributing to the resulting plasma insulin levels. The pancreas, and particularly islets, are densely vascularized, and islet vasculature structure and density can affect the ability of the secreted insulin to reach the blood stream [23]. Although the degree of vascularization (% islet area occupied by CD31-positive cells) was similar between the strains, we cannot exclude differences in vessel structure between WSB and B6 mice. Islet endothelial cells lie in the inner part of the blood vessels, which are covered with pericytes. Nutrient and hormonal signals for insulin secretion reach the islets via the blood and subsequent passage through the endothelial cells [23]. Thus any blockage of these signals from the endothelial cells, such as increased pericyte density or reduced fenestrations/pores in the endothelial cells, could affect the passage of molecules to the β-cells, and thus the amount of insulin secreted or the ability of the secreted insulin to reach the blood, regardless of the vascular density. Pancreatic islets are densely innervated, and neuronal signals can modulate insulin secretion *in vivo* [32]–[34]. Many hormones are also known to affect insulin secretion are removed when islets are studied *in vitro* [30], [35]. In addition, hepatic extraction of insulin, which is secreted into the portal vein, can affect the amount reaching the peripheral circulation [36], [37]. Future studies will be required to more accurately measure secretion *in vivo* accounting for differences in insulin sensitivity and then to determine the mechanism by which insulin secretion is dampened *in vivo* in WSB mice.

Some potential caveats to these studies should be noted. Although many studies have reported an increase in β-cell mass with high fat feeding, e.g. [38], [39], we did not find an increase in β-cell mass in high fat-fed versus chow-fed B6 mice. Islet sizes clearly increased from 4 to 20 weeks of age, but this was similar regardless of diet. The reasons for this are unclear, however may relate to the fact that the mice in our studies were fed the high fat diet from weaning, as most prior studies do not commence high fat feeding until 6–8 weeks of age or later. Thus perhaps high fat diet consumption during post-natal pancreatic growth alters epigenetic programs or compensatory mechanisms such that β-cell mass did not increase in the same way as would be observed if the mice were changed to a high fat diet as adults.

In summary, we have found that WSB mice have interesting diabetes-related phenotypes that are not widely studied, including reduced pancreatic growth, and markedly increased insulin secretion *in vitro*. The molecular bases of these phenotypes are incompletely understood. Therefore, genetic analysis of WSB mice to determine the causative factors is likely to shed important insight into β-cell biology and T2D risk.
